# Drivers of the Growing Water, Carbon and Ecological Footprints of the Chinese Diet from 1961 to 2017

**DOI:** 10.3390/ijerph17051803

**Published:** 2020-03-10

**Authors:** Yiyi Cao, Li Chai, Xianglin Yan, Yi Liang

**Affiliations:** 1 International College Beijing, China Agricultural University, Beijing 100083, China; 2017314060502@cau.edu.cn (Y.C.); yanxianglinbnu@163.com (X.Y.); 2 Chinese-Israeli International Center for Research and Training in Agriculture, China Agricultural University, Beijing 100083, China; 3 College of Science, China Agricultural University, Beijing 100083, China; Cedricly1736@gmail.com

**Keywords:** water footprint, carbon footprint, ecological footprint, food consumption, China

## Abstract

In the past decades, food consumption in China has undergone a rapid increase and a significant structure transition, as a result of population growth and economic development. The food system is increasingly threatening the environment by depleting water resources, deteriorating water bodies, aggravating climate change, degrading ecosystems, etc. It is significant to understand how food consumption affected the environment and how its impacts were driven in the historical period. This study reveals the environmental impacts of China’s food system from 1961 to 2017 from a consumption perspective by assessing water, carbon, and ecological footprints. The logarithmic mean Divisia index method was used to examine the drivers of the growing environmental footprints. The assessment results show that all three environmental footprints have had a drastic increase of more than two times during the studied period, which indicates the high environmental pressure posed by food consumption. We also found that, before the 1980s, the main driving forces of the increasing footprints were population and per capita energy intake. From 1984, the diet pattern started to take a positive effect and then became the dominant driver of the growing environmental footprints after the end of the 1990s.

## 1. Introduction

China’s economy has achieved a great expansion in the past decades. Both population and living standards have increased. The rapidly growing wealth and the urbanization are causing an obvious diet transition in China [1]. Chinese diet preference had shifted from starchy foods, namely tubers and cereals, to protein foods, especially red meats, which aggravates environmental pressure. A large amount of natural resources is required in the life-cycle production of animal-based foods, especially in growing forage crops [2,3]. For example, producing 1 kg of beef consumes an amount of water equivalent to the 75% of daily water requirement of a person [4]. Livestock also contributes a large proportion of global carbon emission [5,6]. An adequate food supply requires the support of enough natural resources. Water resources, for instance, are already scarce in some regions because of agriculture production [7]. The scarcity of natural resources accompanied by the rising need for food is challenging sustainable development [8].

Food production can cause various negative environmental impacts. The water, carbon, and ecological footprints are currently the three most concerned indicators when investigating the environmental impacts of the food system [9,10]. The water footprint (WF) is a useful indicator that can reveal the life-cycle water occupation in producing food [11]. An upward trend of water footprint of China’s food consumption has been proved by Zhang et al. [12]. Although population growth is a great contributor to this growing water footprint [13], the effect of diet transition should also be paid attention to. Researchers claimed that Chinese diet is transiting to a higher quality but meanwhile more water resource is in demand to meet the upgraded diet [14]. Besides consuming water, food system also produces a large amount of greenhouse gases [15]. Carbon footprint (CF) can be used to indicate the direct and indirect greenhouse gas emissions in food system [16]. Compared to the U.S., China has a higher intensity of carbon footprint in food production [9], so China should focus more on the carbon emission from food system. A diet transition to the preference of animal-based foods has led to a rapidly increasing of carbon footprint in China. If a sustainable diet is adopted, e.g., eating more plant-based foods, the carbon emission can reduce by up to 75% [17,18,19]. Kim et al. (2018) proved the climate benefit of adopting a vegetarian diet, and their results emphasized the necessity of reducing red meats consumption for climate action [20]. Besides water shortage and global warming, another concern in China’s food system is the drastically increasing ecological footprint [21,22]. The ecosystem is always a necessity for food production. Although the ecological resources can satisfy the current requirement of China’s food production, China’s ecosystem will be greatly threatened by the diet transition, i.e., eating more animal-based foods [23].

Food system needs to be kept within environmental limits. Food’s environmental impacts can be reduced from producers and consumers [24]. Springmann et al. claimed that only making efforts from the production side, e.g., increasing yield and improving technology, is not enough to keep a food system within environmental limits, so food must be consumed in a sustainable way [25]. Davis et al. called for less impactful diets to feed humans without posing any further pressure on environment [26]. Thus, understanding the environmental impacts in food system from consumption side is essential to achieve sustainability goals. Adopting a sustainable diet is considered to be an effective way to mitigate the environmental pressure from food consumption [27,28]. He et al. (2019) pointed out that the changing food habits in China are becoming a challenge to environmental sustainability [27]. Environmental health and human health can co-exist when people follow dietary guidelines [28] and reduce the consumption of animal-based foods with high environmental footprints [18,29,30].

Investigating how food consumption places pressure on the environment is of significance to help policymakers in adjusting food supply structure and guiding people’s diet, thereby achieving a sustainable food consumption. This study quantifies the water, carbon, and ecological footprints of food consumption in China from 1961 to 2017. The logarithmic mean Divisia index (LMDI) method is then used to decompose the change of footprint into three driving factors, namely, population, per capita energy intake, and diet pattern. We reveal the environmental impact of food consumption and examine its driving factors in the historical period.

Although there have been several studies have focused on the environmental footprint of China’s food consumption, this study makes some further contributions by (1) adopting more recent data in FAO food balance sheet (until 2017), (2) examining the drivers of three highly-concerned footprints (water, carbon and ecology), and (3) identifying the crucial years when each driver started to take effect and when it became predominant. These contributions can provide us with valuable implications when making decisions in the future.

## 2. Methodology and Materials

### 2.1. Food Consumption Data

We derived the data of China’s food consumption during the period from 1961 to 2017 from the Food Balance Sheets (FBS) that were compiled by Food and Agriculture Organization of the United Nations (FAO) [31]. In FBS, the food data were collected from each country at the national level, and then adjusted to ensure material is balanced, i.e., consumption plus export equals to production plus import. For assessing the environmental footprint, we categorized the foods in FBS into 19 groups, namely, maize, rice, wheat, other cereals, soybean, other pluses, nuts & seeds, roots & tubers, fruits, vegetables, vegetable oils, sugar, beef, pork, mutton and lamb, poultry, aquatic products, eggs, and dairy products.

### 2.2. Water Footprint (WF) of Food Consumption

In this study, we adopted the concept from water footprint website [32]. Water footprint measures humanity’s appropriation of freshwater in volumes of water consumed and polluted. To measure the total WF of food consumption in China, the direct and indirect water use during the lifecycle of foods are included. Three water footprints are considered, namely green water, blue water and grey water. Green water means the rainwater required by plants [33]. Blue water refers to surface runoff and ground water [34]. Grey water is the freshwater required to dilute polluted water [35]. The water footprint intensity of each food in this study was obtained from the database build by Mekonnen and Hoekstra [36,37].

Water footprint can be calculated using the following equation:

(1) $$\mathrm{WF}=\sum \left({\mathrm{WF}}_{i}\times {\mathrm{Foodintake}}_{i}\right)$$

where WF is the total water footprint, WF_i_ indicate the water footprint intensity for food item i (WFI contains green, blue and grey water), and Foodintake_i_ refers to the consumption amount of food item i.

### 2.3. Carbon Footprint (CF) of Food Consumption

Carbon footprint measures the total amount of greenhouse gases (GHG) emissions that are accumulated over the lifecycle of products [38]. The Intergovernmental Panel on Climate Change (IPCC) proposed the global potential warming that is measured in CO_2_ equivalent to assess GHG emissions [39]. The coefficient of CF was derived from the footprint database in double food—environmental pyramid model [40].

CF can be assessed using the following equation:

(2) $$\mathrm{CF}=\sum \left({\mathrm{CF}}_{i}\times {\mathrm{Foodintake}}_{i}\right)$$

where CF is the total carbon footprint and CF_i_ is the intensity of carbon footprint for food item i.

### 2.4. Ecological Footprint (EF) of Food Consumption

Ecological footprint is designed to assess human demand on the environment [41]. EF accounts for all kinds of ecological appropriation (including arable lands, forests, building sites, etc.) and converts them into the same measurement unit, i.e., productive land hectare. The EF coefficients of foods in this study were obtained from DFEP database [40].

The total EF of food consumption in China is:

(3) $$\mathrm{EF}=\sum \left({\mathrm{EF}}_{i}\times {\mathrm{Foodintake}}_{i}\right)$$

where EF is the total ecological footprint and EF_i_ is the intensity of ecological footprint for food item i.

### 2.5. Environmental Footprint Intensity

Table 1 lists the environmental footprint intensity for each food group used in this study. According to the original source of these intensities [36,37,40], the assessment boundary is from farm to farm gate, i.e., the environmental footprints occur in stages of transportation, storage, distributing, etc. are not considered in this study.

### 2.6. Logarithmic Mean Divisia Index(LMDI)

To quantify the contribution of each driving factor, we used the logarithmic mean divisia index model [42] to conduct a decomposition analysis. The total environmental footprint is the product of four parameters as shown in below:

(4) $$F=\sum {P\;\times \;E\;\times \;D}_{i}{\;\times \;I}_{i}$$

where F indicates the environmental footprint, including water, carbon and ecological footprints; P is population; E is the per capita energy intake and can indicate whether people obtain enough calories to avoid hunger; D_i_ is the proportion of food item i to total calorie intake (diet pattern), and therefore can indicate the transition of people’s diet preference; I_i_ is the footprint intensity of food i.

This study is from consumption-side perspective, so we assumed that the footprint intensity remains unchanged; this is saying that footprint intensity has no contribution to the change of environmental footprint. Therefore, the change of environmental footprint (F′ − F) can be decomposed into three driving factors, i.e., population, per capita energy intake, and diet pattern, which can be expressed by the following equations:

(5) $$F^{\prime }-F=\Delta F=g\left(\Delta P\right)+g(\Delta E)+g\left(\Delta D\right)$$

(6) $$g\left(\Delta P\right)=\sum \frac{F_{i}^{\prime }-F_{i}}{\ln\left(F_{i}^{\prime }\right)-\ln\left(F_{i}\right)}\ln\left(\frac{P^{\prime }}{P}\right)$$

(7) $$g\left(\Delta E\right)=\sum \frac{F_{i}^{\prime }-F_{i}}{\ln\left(F_{i}^{\prime }\right)-\ln\left(F_{i}\right)}\ln\left(\frac{E^{\prime }}{E}\right)$$

(8) $$g\left(\Delta D\right)=\sum \frac{F_{i}^{\prime }-F_{i}}{\ln\left(F_{i}^{\prime }\right)-\ln\left(F_{i}\right)}\ln\left(\frac{D^{\prime }}{D}\right)$$

## 3. Results and Discussion

### 3.1. The Diet Change of Chinese Residents from 1961 to 2017

**Figure 1:**
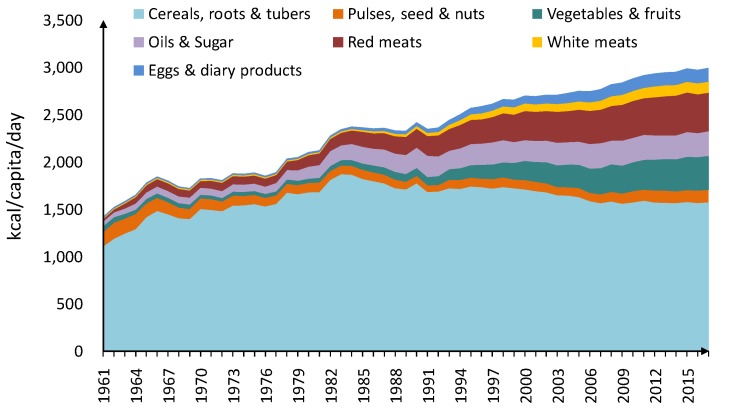
The change of food consumption from 1961 to 2017.

The daily per capita calorie intake is used to measure people’s food consumption. It increased from 1421 kcal in 1961 to 2950 kcal in 2017 as shown in Figure 1. At the same time, the diet pattern changed. In 1961, the main components of Chinese residents’ diet were those starchy foods such as cereals and tubers, which contributed 78% of energy intake, while white meats were the least consumed (only 13 kcal per day). There had been an appropriate increase in animal-based foods such as meats, eggs and dairy products since 1983. By 2017, the per capita calorie intake of red meats and vegetables & fruits, had increased by 16 times and nine times, respectively. The intake of cereals, roots, and tubers went down to half of total daily calories intake. It can be seen that, with the growing income, people’s consumption center was gradually turning to animal-based foods which is rich in protein. Noticeably, cereals, roots and tubers remained as an important source of calories for Chinese and its proportion of daily calorie intake still ranked first.

#### 3.1.1. Water Footprints Embodied in Food Consumption from 1961 to 2017

**Figure 2:**
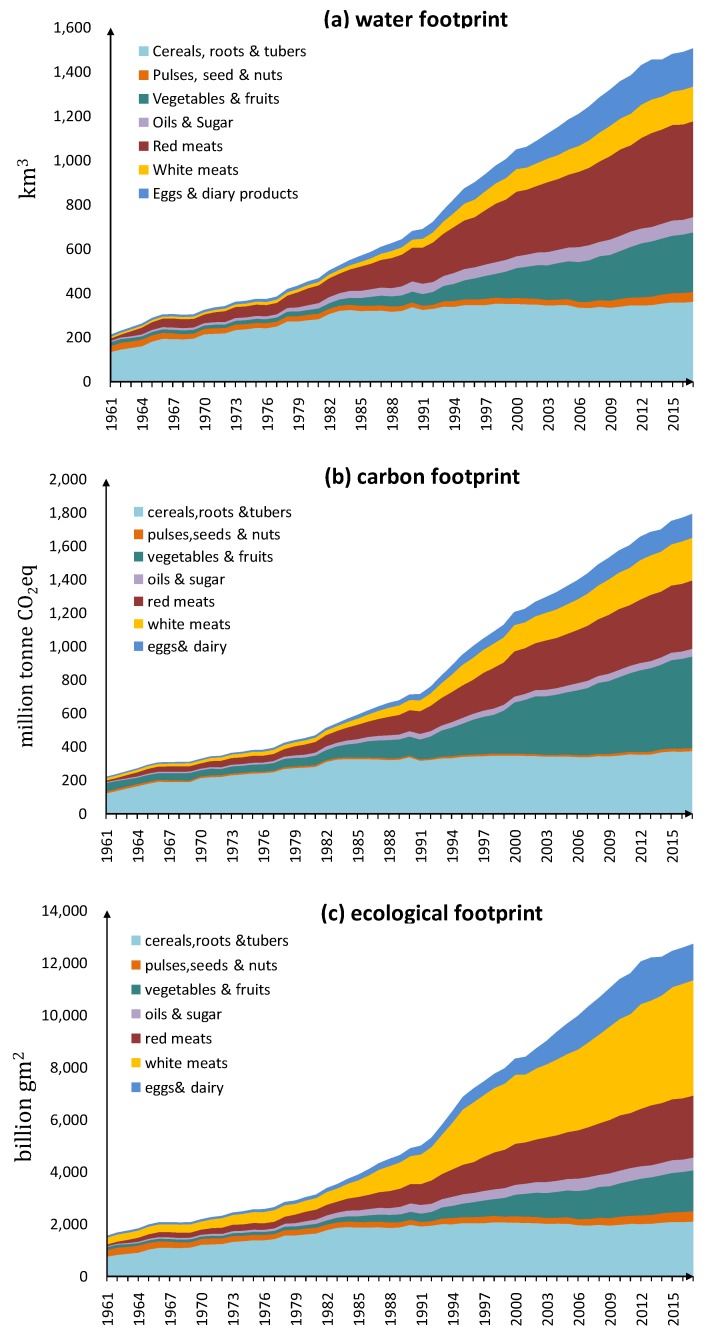
Water, carbon, and ecological footprints of China’s food consumption from 1961 to 2017. (**a**) water footprint; (**b**) carbon footprint; (**c**) ecological footprint.

**Figure 3:**
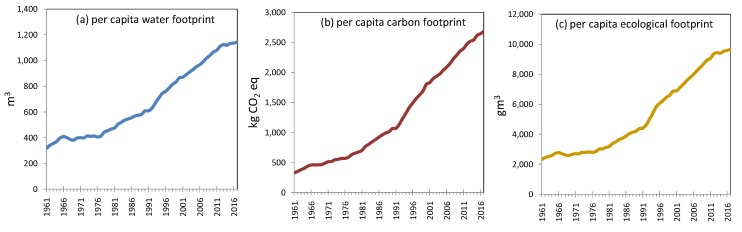
Per capita water, carbon, and ecological footprints of China’s food consumption from 1961 to 2017. (**a**) per capita water footprint; (**b**) per capita carbon footprint; (**c**) per capita ecological footprint.

We assessed the change of virtual water embodied in the consumption of seven categories of food, as shown in Figure 2a. Per capita water footprint of China’s food consumption is on the rise as shown in Figure 3a; it increased from 316 m^3^ in 1961 to 1144 m^3^ in 2017. As shown in Figure 2a, in 1961, cereals had the largest water footprint with around 130 cubic kilometers. The total water footprint was 211cubic kilometers. Since 1961, the water footprint of other categories of food had also been increasing rapidly, the most obvious of which were red meats, vegetables and fruits. The water footprint of the consumption of cereals, roots and tubers had remained stable. In 2017, the water footprint of red meat consumption was the highest, at 424 cubic kilometers, because red meats contain more virtual water than cereals, while Chinese residents are consuming more red meats. Ranked second is cereals with 297 cubic kilometers. The water footprint of consumption of vegetables & fruits reached 250 cubic kilometers. In 2017, the total water footprint of food consumption was 1454 cubic kilometers. Water is becoming an increasingly important energy source for food consumption. Red meats have replaced cereals to become the main culprit of water shortage.

#### 3.1.2. Carbon Emission Embodied in Food Consumption

The trend change of the carbon footprint of China’s food consumption is similar to that of water footprints, with an overall upward trend, as shown in Figure 2b. From 1961 to 2017, per capita carbon footprint increased from 327 kg CO_2_ equivalents to 2681 kg CO_2_ equivalents. In 1961, the consumption of cereals produced the most carbon emission, with 108 million tonnes of CO_2_eq, and the total carbon emission in that year was 219 million tonnes of CO_2_eq. Around 1981, the carbon emission from cereals began to stabilize, while the carbon emissions from other foods began to increase significantly. The most obvious changes were seen in animal-based foods, vegetables and fruits. Carbon emissions from red meats consumption increased from 9.5 million tonnes of CO_2_eq in 1961 to 396 million tonnes of CO_2_eq in 2017; carbon emissions from vegetables and fruits increased from 51.5 million tonnes of CO_2_eq in 1961 to 500 million tonnes of CO_2_eq in 2017. In 2017, the carbon emission from food consumption was 1683 million tonnes of CO_2_eq, of which the consumption of fruits and vegetables was the most, followed by the consumption of red meats, which was 395 million tones of CO_2_eq. However, the carbon emissions from the consumption of beans remained stable from 1961 to 2017, and were far smaller than meats.

#### 3.1.3. Ecological Footprints of Food Consumption

The ecological footprint of Chinese residents’ food consumption is shown in Figure 2c. Overall, the ecological footprint of food consumption is increasing year by year. In 1961, the main factor causing pressure on ecological carrying capacity was the consumption of cereals, which was 531 billion gm^2^ out of the total amount of 1544 billion gm^2^. Since 1981, the ecological load-bearing pressure caused by cereals consumption has been stable, but the ecological footprint of other kinds of food consumption has started to increase significantly, while the ecological footprint of bean food consumption has been stable. The most obvious signs of growth are animal-based foods. In 2017, the ecological footprint for food consumption was 12 thousand billion gm^2^. Among them, the ecological footprint of vegetables and fruits was 1.4 thousand billion gm^2^; red meats consumption was 2.3 thousand billion gm^2^; white meats consumption even reaches 4 thousand billion gm^2^. Compared with water footprint and carbon footprint, white meats contributed a larger proportion of the total ecological footprint.

### 3.2. The Contribution of Three Drivers Ofenvironmental Pressure

**Figure 4:**
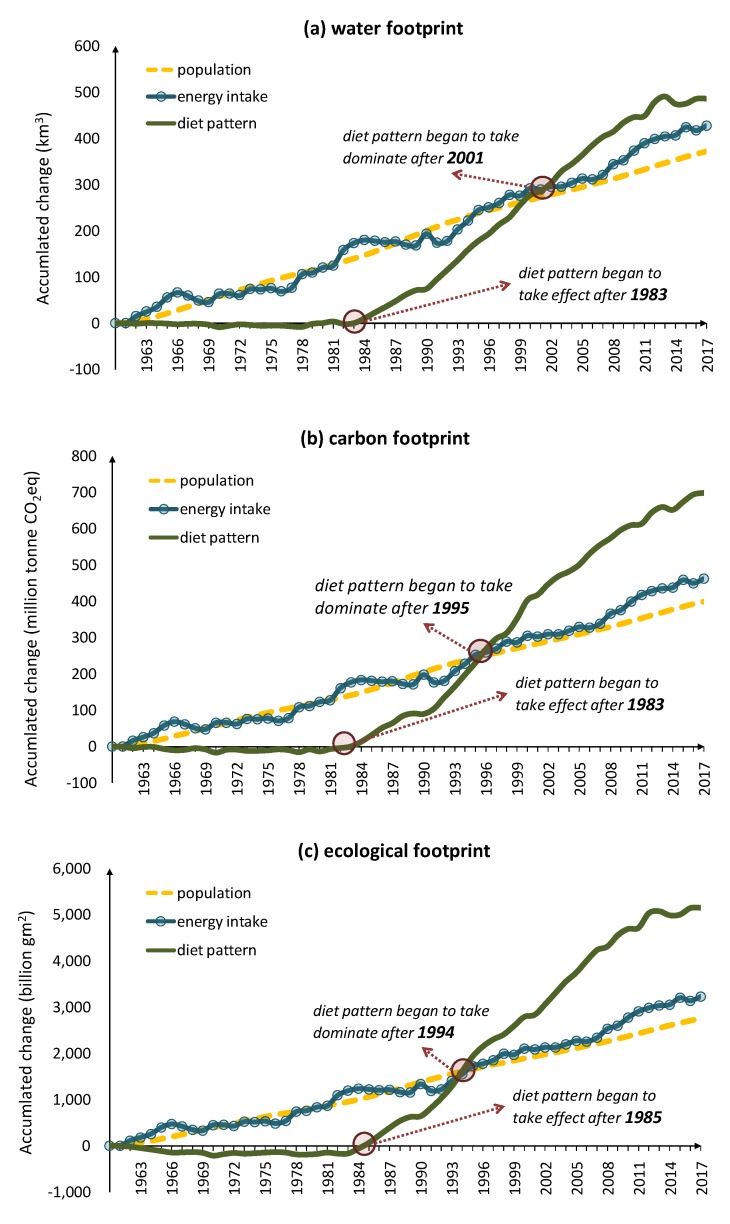
Drivers of water, carbon, and ecological footprints of China’s food consumption from 1961 to 2017. (**a**) water footprint; (**b**) carbon footprint; (**c**) ecological footprint.

Figure 4a shows the contribution of population, energy intake, and diet pattern to water footprints. During the most of period, the increase of population, energy intake per capita, and the transition of diet pattern positively contributed to the increase of water footprints. Before 1983, the contribution of diet pattern was close to zero because the diet pattern during that period almost unchanged. After 1983, the newly changed diet pattern in China began to consume more water than before. Before 1999, the contribution of population was the highest while that of diet pattern was the lowest, but since 1999, the contribution of people’s energy intake and diet pattern both have exceeded that of population and by 2001, Chinese diet pattern has contributed the most to water footprint.

A similar pattern can be observed for carbon footprint and ecological footprint as shown in Figure 4b,c. The diet pattern of China started to contribute to carbon footprint from 1983 and to ecological footprint from 1984. Before 1995, the population in China contributed the most but after 1995, population has become the least important driver of environmental footprints while the diet pattern has become the biggest contributor. By 2017, the contribution of the diet pattern in China to all environmental footprints assessed has been far more than other drivers.

## 4. Discussion

From 1961 to 2017, the per capita energy intake in China increased by more than two times. At the same time, the food consumption was changing from cereals-preferred to meats-preferred, especially red meats. Such a food consumption pattern indicates the rapid improvement of residents’ living standards, but at the same time, it also aggravates the issue of degrading environment. China is rich in natural resources, but the per capita availability of resources is very low. Therefore, the environmental problems caused by the transformation of China’s food consumption are severe. The current resource consumption rate has exceeded the increasing rate carrying capacity of the environment [43,44]. We assessed changes in water, carbon, and ecological footprints for different categories of food. We found that the environmental footprints corresponding to food consumption are increasing rapidly. Although cereals are still the main sources of calories, they are no longer the main factors of environmental pressure. The main consumption of water resources is red meats; the main consumption causing carbon emissions is vegetables & fruits; the main consumption causing ecological area carrying pressure is white meats. These three categories of foods are also the main categories of future diet patterns. White meats are always encouraged by health diet guidelines as a source of protein, but we need to be aware of its potential high ecological footprint. Plant-based foods indeed have lower carbon intensities than meats. For instance, the carbon intensity is 0.93 kg CO_2_eq kg^−1^ for vegetables, while that of meats ranges from 3.41 kg CO_2_eq kg^−1^ of poultry to 31.36 kg CO_2_eq kg^−1^ of beef. Vegetables have occupied a relatively large proportion in the diet of Chinese residents. In 2017, the total consumption of vegetables is 377 kg per capita while that of meats is only 59 kg per capita. This is the reason why vegetables contributed to a large part of the carbon footprint. Still unhealthy, the current Chinese diet consumes too much red meat. For example, in 2017, the daily red meat intake is 163 g per capita, which is much higher than recommends from China’s dietary guidance (50–75 g per day). Therefore, we can conclude that if Chinese residents eat less meats, especially red meats, and more low-CF foods, the food consumption will be more sustainable, the carbon footprint will decrease as well. It is necessary to improve the residents’ environmental awareness, and encourage them adopt a sustainable and balanced diet.

In order to find out which factors cause the greatest environmental pressure in the structure of population, energy intake, and food consumption, this study used the LMDI method to decompose the change of water, carbon, and ecological footprints. The results show that, in the 1980s, the pressure of food consumption on the environment mainly came from the increases of population and per capita energy intake, while the environmental pressure of food consumption in China today mainly comes from an unsustainable diet pattern. Chinese residents now prefer to eat more those foods with high environmental footprints, while the foods with lower environmental footprint are out of favor. According to the planet healthy diet proposed by Lancet, people’s diet can be transformed into one that can supplement enough nutrition without causing excessive body load while reducing environmental pressure, but it is very difficult to restrict people’s diet, so the improvement of technology is still necessary. Although the per capita energy intake is already 3000 kcal per day (2017), obesity and undernutrition still co-exist in Current China, as a result of the uneven distribution of food and food waste. We suggest the government to pay attention to this issue and encourage people to have a healthy diet (e.g., reducing calorie intake for obesity people) and reduce food waste. This can help to reduce the per capita energy intake and thereby reduce the environmental footprint. In addition, trade expansion can also be taken into consideration. China’s “Belt and Road” plan intends to import high demand cereals from the cooperative countries. According to the principle of comparative advantage, it can save resources and meet the domestic demand for food to the maximum extent. As for the environmental impacts of the transportation, currently the consensus is that countries can pay an extra tax to correct the externality. But it’s actually hard to implement without a global government. Moreover, it’s necessary to consider the local industry under global trade, accounting for possible unemployment and competition. So, we need to weigh the loss of GDP against the benefit of trade. In this case, future assessment is needed to compare the net social impact of the excess food production and transportation.

China’s population may potentially continue to grow in the next decade, especially with the implementation of the two-child policy. Thus, the only effective and sustainable route to mitigate the environmental pressure from food consumption is to adopt an environmental-friendly diet, e.g., eating less red meat. This would be beneficial for both human’s health and environment to reduce red meats and make up the calorie loss using white meats and vegetables, because: (1) China’s dietary guidance suggests people to intake more vegetables and claims that white meats (especially fish) are more healthy than red meats; (2) white meats and vegetables contain lower environmental footprints than red meats (as shown in Table 1). As for the protein loss, it can be made up by white meats and legumes. White meats and legumes contain lower environmental footprints than red meats and are encouraged by China’s dietary guidance due to their high contents of protein. The environmental impact of above foods will be tolerable if they are ingested in a moderate and sustainable way. Future research is needed to compare the footprints of different diet patterns intake in order to propose a sustainable diet with certain proportion of different foods. The environmental footprint also can be further lowered by reducing the calorie intake. The per capita energy intake in 2017 already reached 3000 kcal per day, so people should be encouraged to reduce food waste and excessive intake; by doing this, per capita calorie intake can be lowered and thereby the environmental footprint can be reduced. The change of diet habit can’t be achieved in a short time, so the change from the production side is more imperative. Efforts from the production-side, e.g., enhancing yield and improving production efficiency, are encouraged to further lower environmental footprint.

## 5. Conclusions

This paper investigates the trend of environmental footprint of food consumption in China from 1961 to 2017 and examines its driving factors. According to China’s historical food consumption data, the per capita calorie intake in 2017 was more than two times than that in 1961; besides, the diet pattern has also undergone a significant transition in which animal-based foods were consumed more. This resulted in an increasingly environment pressure and a great challenge to sustainability. In the past decades, the water, carbon, and ecological footprints of food production have risen by nearly three times. The main contributor to environmental footprint was cereals in 1961 and then shifted to animal-based foods in 2017. The current Chinese diet pattern is imposing environmental pressure.

We used the LMDI method to identify the main drivers of growing environmental footprints, including population, per capita energy intake, and diet pattern. We found that, before the 1990s, the population had always been the main driver of increasing environmental footprint, but from the 1980s, the diet pattern started to take effect. After the end of the 1990s, the diet pattern exceeded population and energy intake, becoming the main reason for the growing footprint. The results of this study highlight the role of diet pattern in environmental footprint, and implicate the necessity and significance of adopting a sustainable diet for China.

## Figures and Tables

**Table 1:** Environmental footprint intensities used in this study.

Food Items	Water Footprint (m^3^kg^−1^)	Carbon Footprint (kg CO_2_eq kg^−1^)	Ecological Footprint (gm^2^kg^−1^)
Beef	15.41	21.36	112.63
Lamb	5.26	10.44	76.00
Pork	5.99	4.19	24.58
Poultry	4.33	3.41	24.50
Dairy	2.32	1.43	30.00
Eggs	3.28	3.23	14.41
Maize	1.05	0.66	7.50
Rice	1.50	2.51	7.80
Wheat	1.62	0.94	10.63
Other cereals	1.50	1.33	8.76
Fruits	1.05	0.67	4.05
Vegetables	0.27	0.93	2.10
Oils	6.25	2.97	43.97
Soybean	2.44	1.00	21.50
Nuts & seeds	2.44	1.00	21.50
Pulses	2.44	1.00	21.50
Roots & tubers	0.56	0.18	3.00
Sugar	0.52	1.35	4.57
Aquatic Products	1.63	3.85	78.25
